# The Patterns of Recurrences in Idiopathic Benign Paroxysmal Positional Vertigo and Self-treatment Evaluation

**DOI:** 10.3389/fneur.2017.00690

**Published:** 2017-12-15

**Authors:** Hyo-Jung Kim, Ji-Soo Kim

**Affiliations:** ^1^Research Administration Team, Seoul National University Bundang Hospital, Seongnam, South Korea; ^2^Department of Neurology, College of Medicine, Seoul National University, Seoul National University Bundang Hospital, Seongnam, South Korea

**Keywords:** vertigo, benign paroxysmal positional vertigo, treatment, recurrence, self-administration

## Abstract

**Background and Objectives:**

Benign paroxysmal positional vertigo (BPPV) recurs frequently. This study aims to determine that each patient with BPPV has a predilection for a specific canal and the type of recurred BPPV can be predicted from that observed during the previous attack.

**Methods:**

The involved side (right, left, and bilateral) and affected canal (posterior, geotropic horizontal, apogeotropic horizontal, anterior, and mixed) were analyzed in 224 pairs of consecutive attacks of BPPV confirmed in 167 patients at the Dizziness Clinic of Seoul National Bundang Hospital from 2003 to 2017. We defined the recurrence when patients had the redevelopment of BPPV at least 1 week after resolution of the previous one.

**Results:**

During the initial attack, the involved canals were posterior in 134 (59.8%), geotropic horizontal in 53 (23.7%), apogeotropic horizontal in 27 (12.1%), anterior in 5 (2.2%), and mixed in 5 (2.2%). The right ear was more commonly affected than the left ear [132 (58.9%) vs. 90 (40.2%)]. Two patients (0.9%) showed bilateral involvements. During the recurrences, the proportions of involved canals and affected side were similar irrespective of those during the former event. Only 24% of the patients showed the recurrence in the same canal on the same side.

**Conclusion:**

The patterns of recurrences are usually discordant in patients with BPPV. Instruction for self-administration of a specific canalith repositioning procedure based on the previous type of BPPV may have a limited efficacy in this frequently recurrent disorder.

## Introduction

Benign paroxysmal positional vertigo (BPPV) is the most common cause of vertigo and is found in 17–42% of patients with vertigo (1). BPPV is caused by dislodged otoconia that enter the semicircular canals (1–3). When there is a change in the static position of the head with respect to gravity, the otolithic debris moves to a new dependent position within the semicircular canals, leading to a false sense of rotation (1). Canalith repositioning procedures (CRPs) can effectively treat BPPV (4–7). CRP results in immediate resolution of BPPV in about 80% of patients after single application, and the success rate increase up to 92% with repetition of the procedure (8). Since CRP differs according to the involved canal, accurate identification of the affected canal is essential for applying an appropriate CRP to each patient with BPPV. By virtue of its relative easiness, CRP may be attempted by the patients. Indeed, addition of self-applied CRP at home was more effective than the CRP alone performed by the clinician (9–11). Since BPPV recurs frequently with an annual recurrence rate of 15–18% (12, 13), self-administration of CRP may be attempted when BPPV recurs if each patient has a predilection for a specific canal, and the affected canal can be predicted based on the involved canal and type of BPPV during the previous event. Until now, few studies have explored the patterns of canal involvement during recurrences of BPPV (12, 14). In this study, we analyzed the involved canal and type of BPPV during the consecutive pairs of attacks in patients with recurrent BPPV. The purpose of this study is to determine whether the patients with BPPV have a tendency to develop the same type of BPPV on the same side during recurrences of BPPV, which would aid in guiding the patients to treat recurred BPPV for themselves by adopting self-administration of an appropriate CRP.

## Materials and Methods

### Subjects

We analyzed 224 pairs of consecutive attacks of BPPV in 167 patients (51 men, mean age = 64 ± 13 years) at the Dizziness Clinic of Seoul National University Bundang Hospital from 2003 to 2017. Of the 167 patients, 41 had more than 2 consecutive attacks (3 attacks in 29, 4 attacks in 8, and 5 attacks in 4), and 57 additional pairs could be constructed from these patients. We excluded the patients with BPPV in association with head trauma or underlying inner ear disorders and thus included only idiopathic or primary BPPV. All patients received neurotological examinations including spontaneous and gaze-evoked nystagmus, horizontal and vertical smooth pursuit and saccades, limb ataxia, and balance function in addition to routine neurologic examinations. Recurrence was defined when the patients redeveloped positional vertigo and nystagmus at least 1 week after resolution of the previous positional vertigo and nystagmus.

### Diagnostic Procedure

Nystagmus was observed without fixation using a video-Frenzel goggle (SLMED, Seoul, Korea) and was recorded binocularly using a video-oculography (SensoMotoric Instruments, Teltow, Germany). While wearing the goggles in a seated position, spontaneous nystagmus was recorded both with and without fixation during the straight-ahead gaze. To induce positional nystagmus, the patients lay supine from sitting (lying down nystagmus) and turned their heads to either side while supine (head turning nystagmus) (15, 16). Patients were also subjected to the right and left Dix-Hallpike maneuvers and the straight head hanging test (17).

The types of BPPV were determined by the typical patterns of nystagmus induced during the positional maneuvers (18). Those were classified into the posterior, geotropic or apogeotropic horizontal, anterior, or mixed. Bilateral posterior canal BPPV was diagnosed when patients showed torsional upbeat nystagmus with the torsional nystagmus beat in the direction of Dix-Hallpike maneuvers, i.e., clockwise (from the patient’s perspective) upbeat nystagmus in right Dix-Hallpike maneuver and counter-clockwise upbeat nystagmus in left Dix-Hallpike maneuver. For secure diagnosis of paroxysmal positional downbeat nystagmus of peripheral origin, we included only those with paroxysmal positional vertigo along with downbeat nystagmus and clear torsional nystagmus beating in the direction of Dix-Hallpike maneuvers and resolution of vertigo and nystagmus with canalith repositioning maneuvers (18, 19). The mixed type of BPPV was diagnosed when more than one canal were involved. The pairs of attacks during the consecutive episodes were classified into *concordant* and *discordant* when the types of BPPVs were identical and different, respectively.

We analyzed the affected canal and type of BPPV during the consecutive recurrences of BPPV.

### Statistical Analyses

The *t*-test or Mann–Whitney *U* test was used to compare the continuous variables (age and interval) between the concordant and discordant groups. Chi-square test was adopted to compare the nominal variables (distribution of the involved canals and affected ear and the sex) between the groups. All the tests were performed using R (version 3.3.3),^1^ and *p* < 0.05 was considered significant.

### Standard Protocol Approvals and Patient Consents

All experiments followed the tenets of the Declaration of Helsinki, and this study was approved by an Institutional Review Board of Seoul National University Bundang Hospital (IRB No. B-1707/409-103). This study was approved by an Institutional Review Board for exemption from the subjects’ informed consent because this study was the retrospective analysis of the medical records.

## Results

The interval from the initial event to the recurrence ranged from 7 to 3,229 days (median = 192 days). Overall, 50% of the recurrences occurred within 6.4 months from the initial attacks.

### Involved Ears

**Figure 1:**
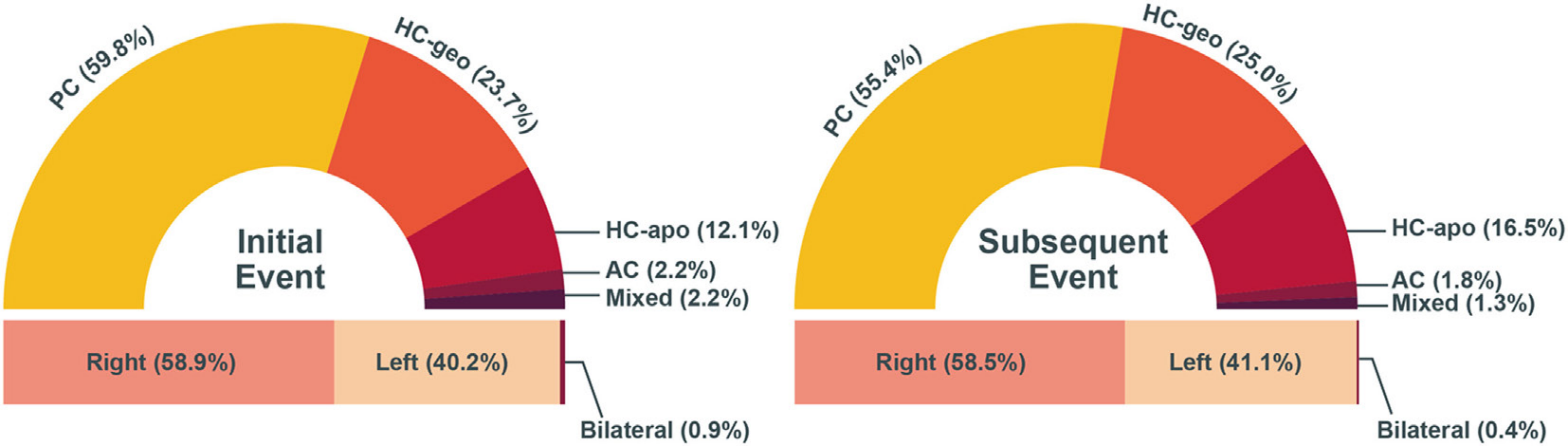
The proportion of involved canal and ear during the initial and subsequent events. There was no difference in the proportion of involved canal and affected side between the events (Chi-square test, *p* > 0.05). AC, anterior canal; HC-apo, apogeotropic horizontal canal; HC-geo, geotropic horizontal canal; PC, posterior canal.

**Figure 2:**
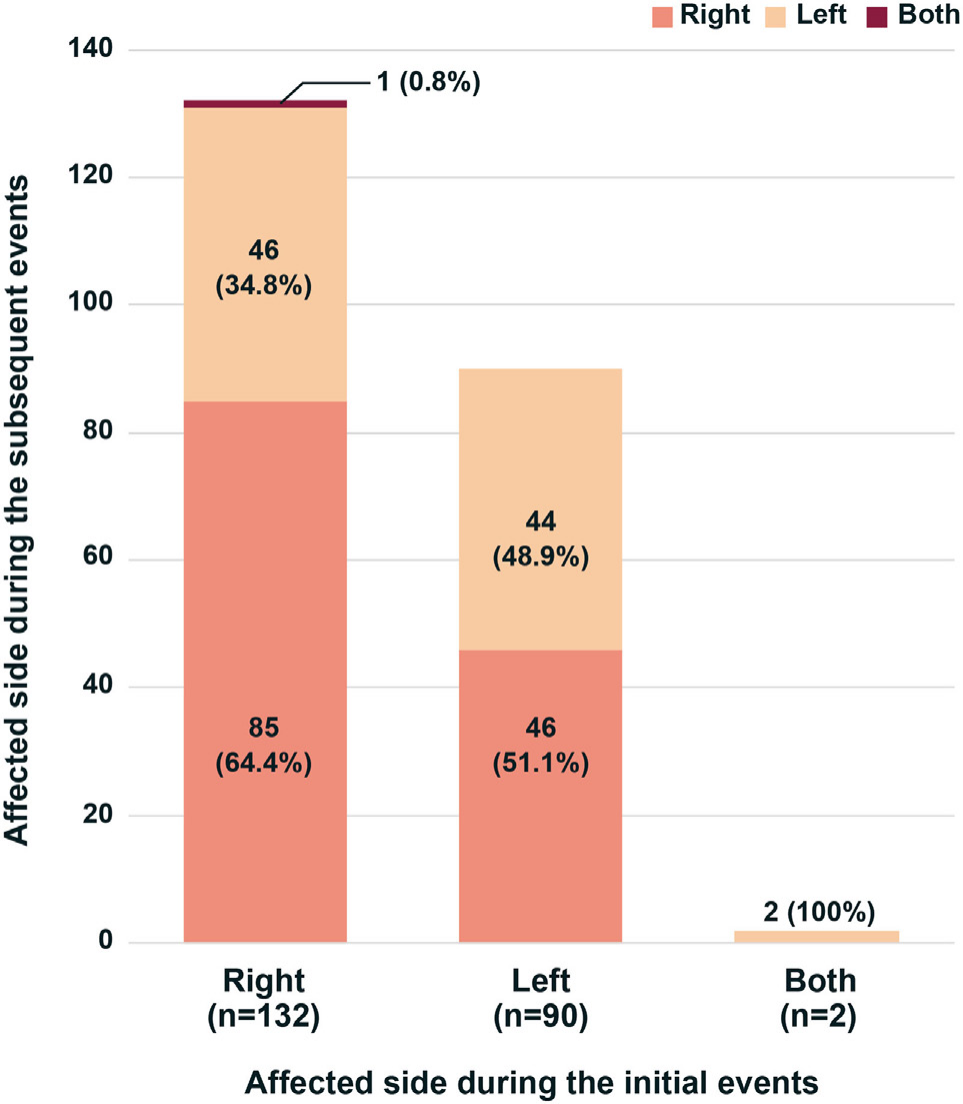
Involved sides of benign paroxysmal positional vertigo during recurrences. Irrespective of the side involved during the initial attacks, the proportion of affected sides was similar during the recurrences (Chi-square test, *p* = 0.123).

During the initial attacks, the right ear was affected in 132 (58.9%), the left ear in 90 (40.2%), and both ears in 2 (0.9%) patients. During the subsequent attacks, the right ear was involved in 131 (58.5%), the left ear in 92 (41.1%), and both ears in 1 (0.4%) patient. There was no difference in the proportion of affected ear between the initial and following events (Figure 1, Chi-square test, *p* = 0.8356). Furthermore, no difference was found in the proportion of the affected side during the following events between the patients with right and left ear involvements during the initial events (Figure 2, Chi-square test, *p* = 0.123). Thus, only 57.6% (129/224) of the patients with a unilateral involvement of the ear during the initial attacks showed an affection of the ear on the same side during the following attacks, which did not differ from a random chance of involving either ear (Chi-square test, *p* = 0.2509).

### Types of BPPV

During the initial attack, the involved canals and types were posterior in 134 (59.8%), geotropic horizontal in 53 (23.7%), apogeotropic horizontal in 27 (12.1%), anterior in 5 (2.2%), and mixed in 5 (2.2%) patients without a difference in the proportion of the types between the patients with right and left ear involvements (*p* = 0.202, Chi-square test). A similar pattern of distribution was observed in the types of BPPV during the subsequent attacks (Figure 1; Chi-square test, *p* = 0.619).

**Figure 3:**
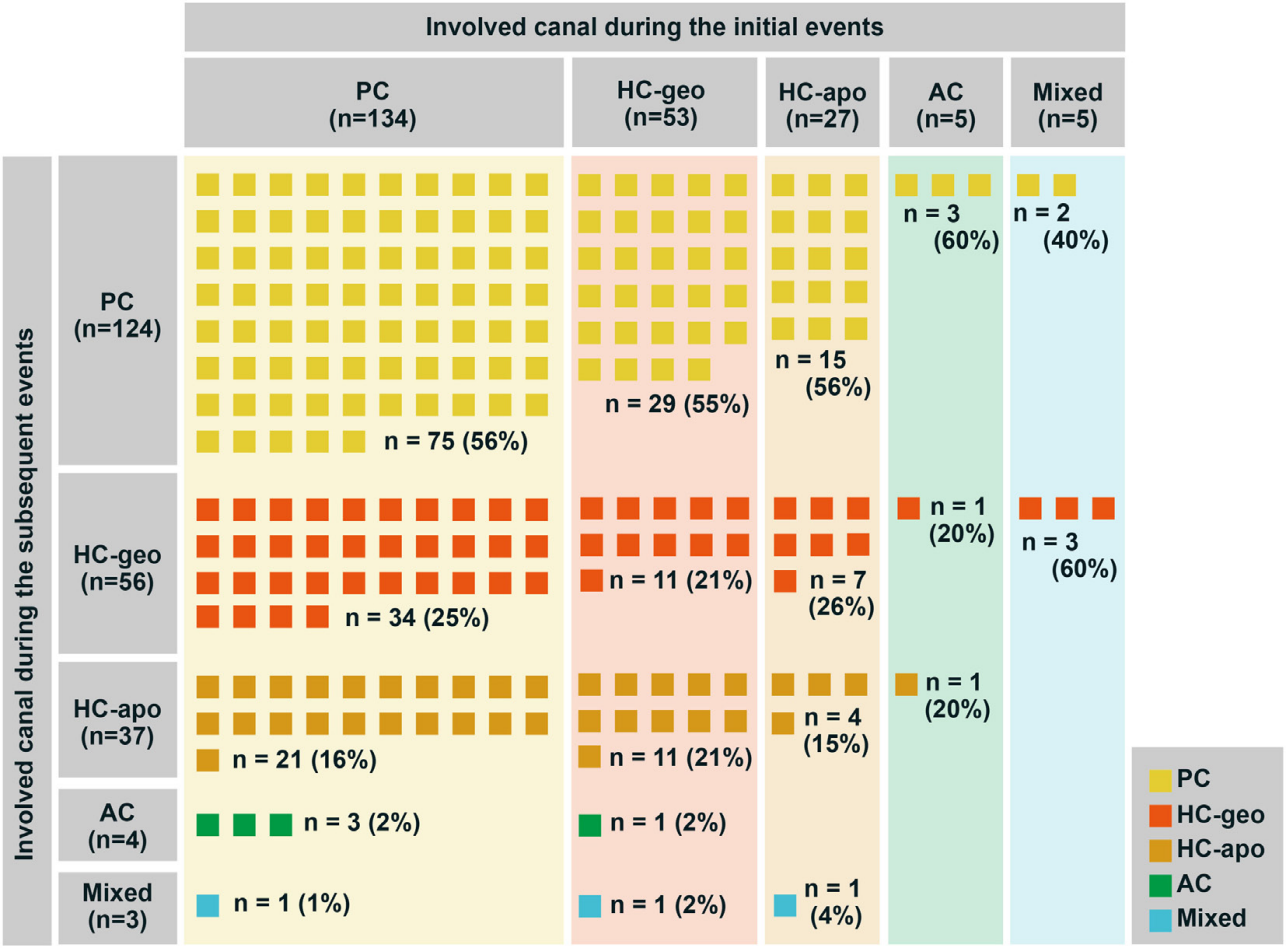
The types of benign paroxysmal positional vertigo (BPPV) during recurrences. Irrespective of the types of BPPV during the initial attacks, the proportion of BPPV types showed no difference during the following attacks (Chi-square test, *p* = 0.9738). AC, anterior canal; HC-apo, apogeotropic horizontal canal; HC-geo, geotropic horizontal canal; PC, posterior canal.

**Figure 4:**
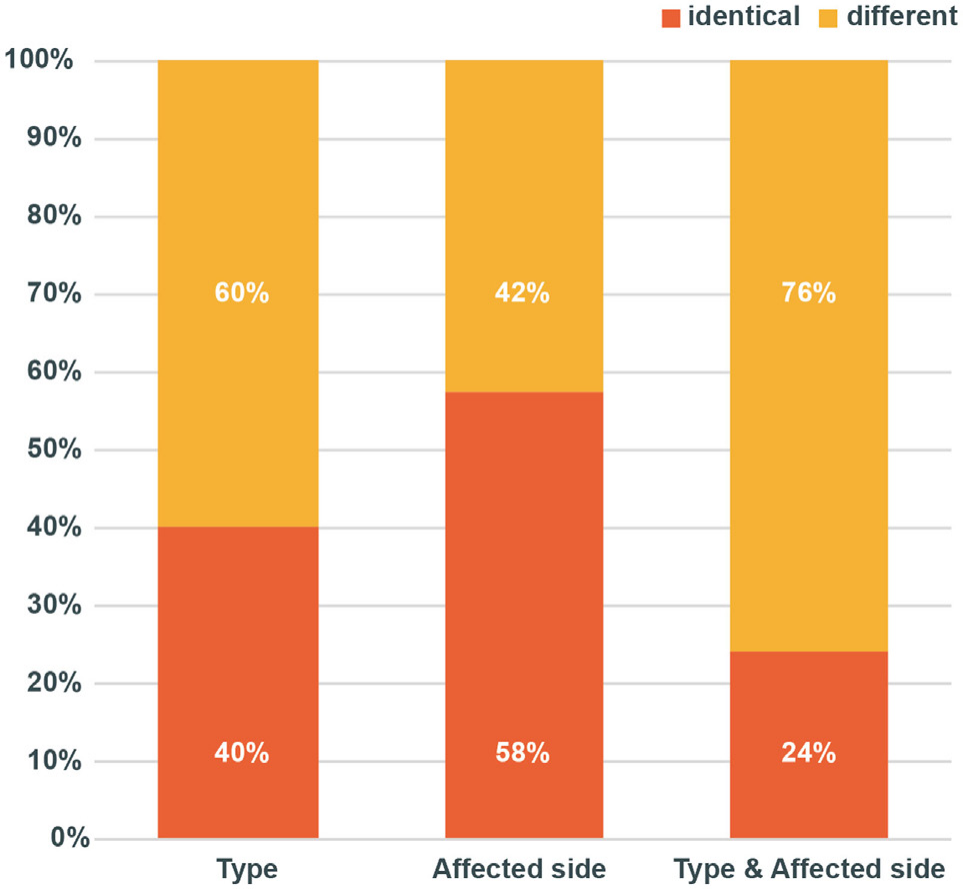
Patterns of recurrences. During the recurrences, proportion of the involved canals (*p* = 0.9738) and affected side (*p* = 0.7966) did not differ from those of the initial event (Chi-square test). Only 24% of the patients showed the recurrence in the same canal on the same side.

Irrespective of the types of BPPV during the initial attacks, the proportion of BPPV types was similar during the following attacks (Figure 3; Chi-square test, *p* = 0.9738). Even when only the three frequent types (posterior canal and geotropic and apogeotropic horizontal canal types) were included for analyses, no difference was found either (Chi-square test, *p* = 0.9419). Thus, only 40% (90/224) of the patients showed the same type of BPPV on either side during the recurrences (Figure 4).

### Patterns of Recurrences

Overall, only 24% (54/224) of the patients showed the same types of BPPV on the same side during the recurrences (Figure 4). Between the concordant and discordant groups, the age (*p* = 0.340, *t*-test), sex (*p* = 0.340, Chi-square test), and the interval between the attacks (*p* = 0.079, Mann–Whitney *U* test) did not differ. Especially, no difference was found in the patterns of recurrences between the pairs with an interval of ≤30 days and those with an interval of more than 30 days.

## Discussion

In this study, only 24% of patients with idiopathic BPPV show the same type of BPPV on the same side during consecutive pairs of BPPV attacks. This finding is consistent with the results of a previous study that found recurrences of posterior canal BPPV in the same canal only in 33% of patients with unilateral idiopathic posterior canal BPPV (20). All these findings indicate a random chance of the subtypes of BPPV when recurs irrespective of the types of BPPV during the previous attack. We may expect involvement of the same canal in recurred BPPV when the interval is short. However, our data showed no difference in the interval between the concordant and discordant groups and no difference in the patterns of recurrences in the pairs with an interval of ≤30 days and those with an interval of more than 30 days.

Since its first introduction for posterior canal BPPV (4, 21), CRPs became available for each type of BPPV and results in successful treatments in 80–90% of patients when applied appropriately (8, 22, 23). Thus, the prerequisite for successful treatments is an accurate identification of the involved canal and type in BPPV. BPPV is a highly recurrent disorder with a 15% recurrence rate per year and a 50% of recurrences within 40 months (12). Since many video clips have been developed and became available on the video-sharing web sites^2^ for easy application of CRPs for each type of BPPV, self-administration of CRP has become feasible in treating recurred BPPV if the patients have the information on the type of BPPV they are suffering from. However, the video clips of CRP placed on the web sites have an accuracy of 64% (24). Thus, the instruction by experts would be more effective for accurate selection and application of CRP for each type of BPPV, and self-administration would be especially effective in patients with recurred BPPV when they have prior instruction on the application of appropriate CRP for the BPPV recurred. Indeed, a previous study demonstrated that patients can treat BPPV by themselves especially when in-person instructions on CRP were provided by an expert (9, 11).

Patients with BPPV bear an average medical expense of US$2,684.74 per individual in the United States (25) and 364 euros in Spain (26). Even in South Korea where the medical expenses are relatively low, individual patient with BPPV spend about US$180 on average for each attack of BPPV. If the type of recurred BPPV can be predicted, self-administration of CRP would be effective in treating BPPV and would greatly reduce the medical costs and social burden related to this highly prevalent vestibular disorder.

The posterior canal has been known to be involved mostly in BPPV (27, 28), and the right ear is more commonly involved than the left one (27, 29). The frequent involvement of right posterior canal has been explained by the right recumbent position preferred by the people during sleep and dependent location of the posterior canal while lying down (30, 31). Thus, patients may attempt the CRPs designed for the right posterior canal BPPV first and then for the left posterior canal BPPV if the first trial fails. However, recent studies have demonstrated that BPPVs involving the canals other than the posterior one are more prevalent than believed previously (32). The proportion of each type of BPPV depends on the interval from the symptom onset to evaluation, and the higher proportion of posterior canal BPPV has been reported in referral-based dizziness clinics that usually deal with the intractable cases (32). Thus, the real prevalence of posterior canal BPPV may be lower than believed, especially during the acute phase. Indeed, right posterior canal was involved in only 37.5% of our patients with BPPV. Thus, we became interested in whether each patient with BPPV has a predilection for a specific type of BPPV and we can predict the type of BPPV when it recurs based on that observed during the previous event.

In this study, the pattern of recurrences was usually discordant in our patients with idiopathic BPPV. Since we could not predict the type of recurred BPPV based on the type observed during the previous attack, education for self-administration of a specific CRP for recurred BPPV may have a limitation in treating this frequently recurrent disorder. For successful self-treatment of recurred BPPV, other ways to predict the types of BPPV should be developed.

## Ethics Statement

All experiments followed the tenets of the Declaration of Helsinki, and this study was approved by Institutional Review Board of Seoul National University Bundang Hospital (IRB No. B-1707/409-103).

## Author Contributions

H-JK acquired and analyzed the data and drafted the manuscript. J-SK conducted the design and conceptualization of the study, interpretation of the data, and revision of the manuscript.

## Conflict of Interest Statement

J-SK serves as an associate editor of Frontiers in Neuro-otology and on the editorial boards of the Journal of Clinical Neurology, Frontiers in Neuro-ophthalmology, Journal of Neuro-ophthalmology, Journal of Vestibular Research, Journal of Neurology, and Medicine. The other author has nothing to disclose.

## Footnotes

^1^https://www.r-project.org/.

^2^http://www.youtube.com.
